# Determination of the bruise degree for cherry using Vis-NIR reflection spectroscopy coupled with multivariate analysis

**DOI:** 10.1371/journal.pone.0222633

**Published:** 2019-09-18

**Authors:** Yuanyuan Shao, Guantao Xuan, Zhichao Hu, Zongmei Gao, Lei Liu

**Affiliations:** 1 College of Mechanical and Electrical Engineering, Shandong Agricultural University, Tai’an, Shandong, China; 2 Nanjing Research Institute For Agricultural Mechanization, Ministry of Agriculture, Nanjing, Jiangsu, China; 3 College of Agriculture, Food and Natural Resources, University of Missouri, Columbia, Missouri, United States of America; 4 Department of Biological Systems Engineering, Washington State University, Pullman, Washington, United States of America; Politechnika Krakowska im Tadeusza Kosciuszki, POLAND

## Abstract

Determination and classification of the bruise degree for cherry can improve consumer satisfaction with cherry quality and enhance the industry’s competiveness and profitability. In this study, visible and near infrared (Vis-NIR) reflection spectroscopy was used for identifying bruise degree of cherry in 350–2500 nm. Sampling spectral data were extracted from normal, slight and severe bruise samples. Principal component analysis (PCA) was implemented to determine the first few principal components (PCs) for cluster analysis among samples. Optimal wavelengths were selected by loadings of PCs from PCA and successive projection algorithm (SPA) method, respectively. Afterwards, these optimal wavelengths were empolyed to establish the classification models as inputs of least square-support vector machine (LS-SVM). Better performance for qualitative discrimination of the bruise degree for cherry was emerged in LS-SVM model based on five optimal wavelengths (603, 633, 679, 1083, and 1803 nm) selected directly by SPA, which showed acceptable results with the classification accuracy of 93.3%. Confusion matrix illustrated misclassification generally occurred in normal and slight bruise samples. Furthermore, the latent relation between spectral property of cherries in varying bruise degree and its firmness and soluble solids content (SSC) was analyzed. The result showed both colour, firmness and SSC were consistent with the Vis-NIR reflectance of cherries. Overall, this study revealed that Vis-NIR reflection spectroscopy integrated with multivariate analysis can be used as a rapid, intact method to determine the bruise degree of cherry, laying a foundation for cherry sorting and postharvest quality control.

## Introduction

Cherry (*Cerasus pseudocerasus*) is one of the most popular fruits because it tastes unique and is small and more like berry than fruit. Meanwhile, it contains sugars, minerals, vitamins and other nutrients [1–2]. Cherry is juicy and prone to mechanical damage and impact when they collide with each other or a hard surface during harvesting, handling, storage, transportation and distribution. In generally, customers pick up cherries and judge its quality by the external attributes such as shape, colour and size [3]. A moderate amount of bruise is a barrier to purchasing desire of consumers instead of price [4], which will lead to postharvest loss and decrease in profits to cherry industry. Moreover, cherry affected by bruises will tend towards fermentation, decay or mildew and infect other non-bruised ones after damage occurrence [5–6]. Therefore, it is necessary to distinguish the bruised cherry from the non-bruised ones and sort them out before sale. However, bruise detection is commonly carried out subjectively by manual inspection with labour cost increased, and some slight bruise having no obvious color change and juices outflow are observed hardly only by naked eyes. Moreover, the efficiency and accuracy will also be reduced greatly after continuous manual inspection. Hence, it’s of great importance to develop a rapid, non-contact detection technique to identify the cherry bruise and determine its bruise degree. By sorting cherry in accordance with its bruise degree, better quality means better price and less food waste increasing profits.

Machine vision technology based on visible imaging system has become widely used for bruise detection in fruits over the past few years [7–9]. However, early changes in bruise area for fruit are difficult to be detected by traditional RGB vision system for part of fruits including cheery [10]. Moreover, inhomogeneous reflection intensity of the spherical fruit also exists in the colour image and leads to inaccurate and ineffective detection in bruise area [11], which limits the application of image analysis techniques. Visible-near infrared (Vis-NIR) reflection spectroscopy technique, which can acquire information about both external defects and internal compounds of the samples, has got more concern given the rising demand for rapid and accurate quality measurement in fruit and vegetable products [12]. A simple identification mode for jujube bruise has been established with NIR spectroscopy [13]. Fungal infections on citrus fruit, skin defects of bi-colored peaches and the content of bioactive compounds in intact tomato have been detected using Vis-NIR reflectance spectroscopy [14–16]. Some researchers have conducted the non-destructive detection about blackspot in potatoes using Vis-NIR and short-wave infrared (SWIR) hyperspectral imaging [17]. A rapid and non-destructive method has been developed to measure flesh colour in clingstone peaches and the internal quality of intact mango based upon Vis-NIR reflectance spectroscopy [18–19]. Vis-NIR technique has been also used to measure the quality of some fruits such as SSC and firmness for pears, apple and blueberries [20–28], astringency in persimmon [29]. Additionally, such technology has been used to predict the pH of fresh chicken breast fillets [30], perform non-invasive assessment of freeze-thaw cod [31], and detect the chlorophyll content in corn leave combining support vector machine (SVM) [32], among other purposes.

Spectroscopic techniques including Vis-NIR spectroscopy have increased in importance for fruit bruise detection coupled with multivariate analysis methods. Bruising with time elapsing on five varieties of apple was compared by supervised classification methods, including SVM, linear logistic regression, neural networks and decision trees [33]. Least square-support vector machine (LS-SVM) model was set up for identifying the subtle bruise of fresh jujube with the effective wavelengths picked up by PCA and SPA combination methods [13]. PCA and radial basis function-support vector machine (RBF-SVM) classification were used in Vis/NIR hyperspectral imaging for detection of hidden bruises image on kiwifruit [34]. Partial least squares (PLS) method and stepwise discrimination analysis were used for data dimensionality reduction and selecting the effective wavelengths in early detection of apple bruises on different background colours using hyperspectral imaging [35]. To compare bruise detection on five varieties of apples, different supervised classification models were set by method of SVM, simple logistic (SLOG), sequential minimal optimization (SMO) and receiver operating characteristic (ROC) analysis [36–37]. Early bruises in apples were detected using hyperspectral imaging, thermal imaging and many different analysis methods including PCA, soft independent modelling of class analogy (SIMCA), linear discriminant analysis (LDA) and SVM [38].

Recently, some studies were focusing on quality control for tart or sweet cherries and blueberries based on spectroscopy techniques. One of them was to detect pits or internal insect infestation in tart cherry using transmittance spectroscopy [39–41]. NIR spectroscopy technique was implemented to detect the internal bruises of blueberries after mechanical impact with different measurement time, and to classify hard blueberries and soft blueberries [42–43]. Moreover, defect detection on sweet cherry, and non-destructive measurement of soluble solids and dry matter content in sweet cherry were operated by applying NIR spectroscopy techniques [44–47]. However, application of Vis-NIR spectroscopy technique to determine the bruise degree of cherries has been not studied in depth for detail classification, which would be investigated in this study.

Collectively, the objectives of this study were to: (1) collect cherry samples and classify them into three groups visually by expertise researcher according to bruise level, (2) investigate the potential of Vis-NIR spectroscopy coupled with multivariate analysis methods to determine the bruise degrees of cherry, (3) explore the latent relationship between spectral property and the SSC and firmness in varying bruise degree of cherries and its chemical composition.

## Materials and methods

### Experimental materials

**Fig 1:**
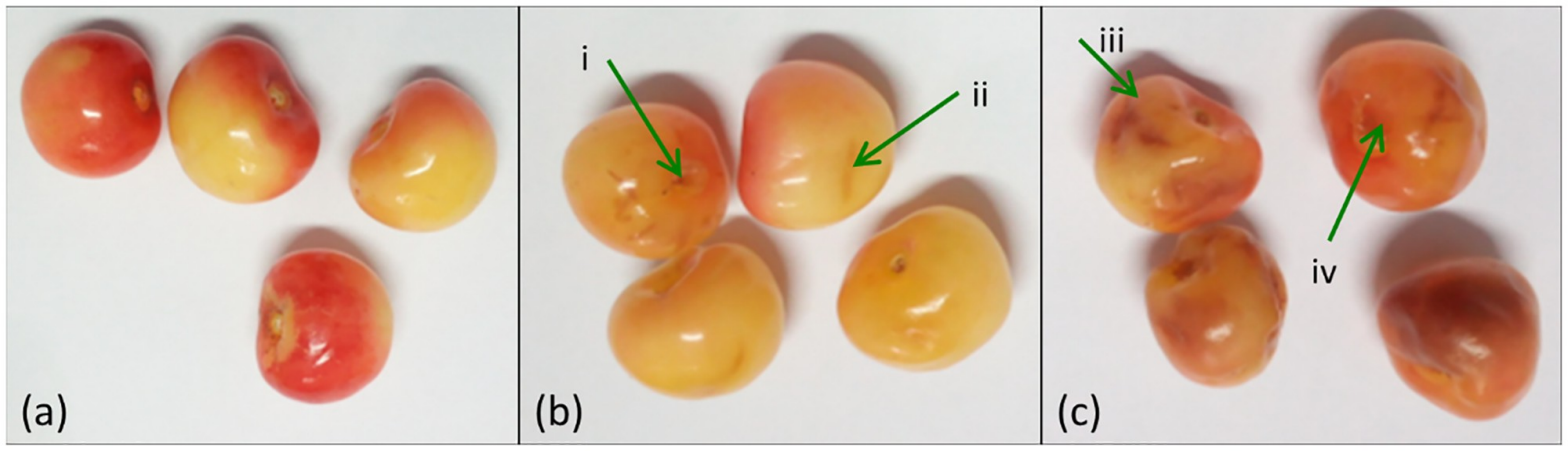
‘Huangmi’ cherry samples in varying bruise degree. (a) Normal; (b) slight bruise; (c) severe bruise.

‘Huangmi’ sweet cherry were purchased in June 2018 from the local Agricultural Market, Taishan District, Tai’an City, Shandong Province, China. According to the size and number of bruises on the surface of cherries as well as consumer acceptance, these samples were classified into three categories followed handpicked carefully: normal, slight bruise and severe bruise [48]. There were 100 samples at each category, and a total of 300 samples were studied in this experiment. As shown in Fig 1(a), there was no visible damage in normal cherries, and these samples are smooth on surface and bright in colour. Slight bruise samples had subtle changes in colour and small scratches in bruise area, such as “i” and “ii” part shown in Fig 1(b). It is difficult to find these slight bruises by customers. In severe bruise samples in Fig 1(c), scratches “iii” and pits “iv” had deepen and enlarged colours in bruise area, even more, colour in these bruise areas changed to brown or dark brown. These bruise cherries could not be accepted by customers, and they should be removed from normal and slight bruise cherries, otherwise, overall profits would grow down due to a fall in sale price of cherry.

### Spectral data acquisition

Spectral data of cherries were acquired by the FieldSpec4 spectrometer (Analytical Spectral Device Company, Boulder, CO, USA), which covers the wavelength range of 350–2500 nm and scan time was 100 ms. Spectral resolution was 3 nm@700 nm and 8 nm@1400/2100 nm. Accuracy and repeatability for wavelength were 0.5 nm and 0.1 nm, respectively. A 15V/75W halogen lamp (Analytical Spectral Device Company, Boulder, CO, USA) was placed on the tripod 50 cm away from the sample as the only light source, and an optical fiber probe in a pistol grid was mounted on a tripod and was vertical to the sample at the distance of 15cm. The experiment was executed in a dark room with uniform incident light at an angle of 45° to the horizontal plane of the sample. Before performing the experiment, the spectrometer needed to warm up about 30 minutes for eliminating the influence of background on the spectral information, and lamp must be on for a while because of spectral stability. Calibration was performed using white reference plate, and then spectral measurement was carried out at three different locations of each cherry with an optical fiber probe. The spectrum data was collected with RS3 software (Analytical Spectral Device Company, Boulder, CO, USA).

### SSC and firmness measurement

After acquiring the spectral data, the physical and chemical indexes of cherry samples were determined rapidly in the postharvest engineering laboratory at Shandong Agricultural University, Tai’an, Shandong Province, China. The firmness of cherries was first measured three times at different locations using GY-1 type fruit hardness tester (Thorpe Ltd, Zhejiang, China) with a 3.5 mm diameter steel probe, and the average value was calculated. And then juice extracted from the cherries was used for SSC measurement (°Brix) with LB20T type refractive digital sugar-meter (Ming Rui Electronic Technology Ltd, Guangzhou, China).

### Multivariate analysis methods

PCA, as a statistical method to analyze and simplify datasets, is usually used to reduce the dimension of datasets and extract feature information [49], and can be defined using the following expression:

$$Y=t_{1}•p_{1}^{T}+t_{2}•p_{2}^{T}+\ldots +t_{K}•p_{K}^{T}+E$$ (1)

Where *Y* is the matrix of spectral samples, *t* is score vector, *p* is loading vector, and *E* is residual matrix. Each principal component (PC) is a linear combination of all original variables, and several PCs are orthonormal and produced in PCA transform. Generally, the first few PCs can reveal most relevant information, and score express their importance. The loadings indicate the contributions of the wavelengths and their extreme values are further indicating optimal wavelengths to be selected [50].

Successive projection algorithm (SPA) is a forward selection method used to select variable wavelength from full spectra of cherry by MATLAB 2011a software (The MathWorks, Inc., Natick, MA, USA). When selecting optimal sets of variables for multivariate calibration classification models in bruise degree, SPA starts with one wavelength, and then incorporates another new one in each iteration until a specified number N of wavelengths is reached. Optimal wavelengths selection aims to select only a few wavelengths which carry the most of useful information. In other words, full spectra are replaced with minimum full spectra for decreasing computation of spectral data. In this study, SPA and the loading values of PCs were used to select optimal wavelengths to reduce data dimensionality [51].

Least squares support vector machines (LS-SVM) classification models were set up with MATLAB 2011a software to accurately identify cherry bruise using the spectral data and the corresponding labelled classes, and its regression model is expressed as follows:

$$y(x)=\sum_{i=1}^{N}\alpha_{i}K\left(x,x_{i}\right)+b$$ (2)

Where *y* is prediction value, *x* is unknown sample, *α*_i_ is Lagrange operator, *K*(*x*, *x*_*i*_) is kernel function, *x*_*i*_ is input vector, *b* is deviation, *N* is sample quantity. Before establishment of LS-SVM models, the data matrix and corresponding labelled class of each spectrum are firstly implemented to divide into a calibration set and a predication set [52]. Here, Kennard-Stone (K-S) method is employed to finish this step [53]. Representative samples which has a large spectral difference (the farther away from the Euclidean distance) is selected to the calibration set, and the rest more similar samples are placed in the prediction set. This will make the calibration set have uniform distribution in the broadest sense. LS-SVM methodology, an optimized version of the standard SVM, is one of supervised learning methods (classes or composition of the samples in the data matrix is involved). It has a wide application for pattern recognition and function estimation. In detail, the Gaussian RBF kernel function was chosen, and the parameters of γ and σ^2^ were optimized by a grid search procedure and 10-fold cross validation. In this paper, normal, slight and severe bruise cherries were assigned dummy grade values of 1, 2 and 3. Then, samples in each class was divided into a calibration set and a prediction set with the ratio of 3:1 by K-S methodology, hence a total of 225 samples were selected as the calibration set, and the remaining 75 samples were used as the prediction set. Finally, performances of LS-SVM models were evaluated with the recognition rate of prediction set as classification accuracy of bruise degree for cherry. Classification and sample number of cherries in varying bruise degree were demonstrated in Table 1.

**Table 1:** Classification and sample number of cherries in varying bruise degree.

Bruise degree	Sample number	Calibration	Prediction	Classes
Normal	100	79	21	1
Slight bruise	100	75	25	2
Severe Bruise	100	71	29	3

### Data analysis and software

The data pre-processing, statistical calculations and analyses were carried out by the ViewSpec Pro 6.2.0 (Analytical Spectral Device Company, Boulder, CO, USA), ENVI4.6 (Environment for Visualizing Images software, Research Systems Inc., Boulder, CO, USA), MATLAB 2011a and the Unscrambler X10.1 (CAMO AS, Oslo, Norway). In detail, LS-SVM models were set up using LS-SVM v1.8 toolbox running on MATLAB R2011a and all graphs were designed by Origin8 SR0 (Origin Lab Corporation, Northampton, MA, USA). All operations were run on a PC installed Windows 7 operating system (Intel(R) Core (TM) i7-6500U @2.50GHz, RAM 8.00GB).

**Fig 2:**
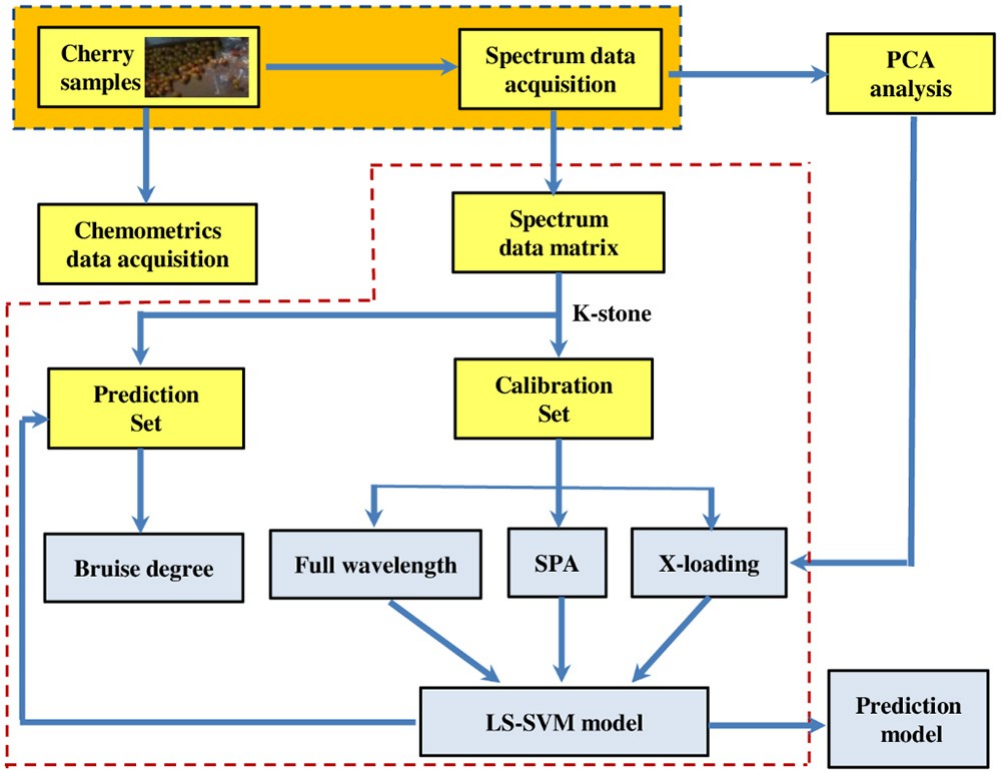
Key steps of the experimental procedure.

In all, key steps for the whole experimental procedure were shown in Fig 2. Firstly, spectral data were acquired by spectra measurement system, and mean reflectance spectra were obtained by using ENVI software. Then PCA method was conducted to make cluster analysis for three categories of cherries with Unscrambler X10.1 software. Then, full spectral data was divided into calibration set and prediction set by K-S method in MATLAB 2011a, and optimal wavelengths were selected with SPA and loadings from PCA, respectively. Furthermore, LS-SVM models were constructed to identify cherry bruise degree.

## Results and discussion

### Spectral profiles

**Fig 3:**
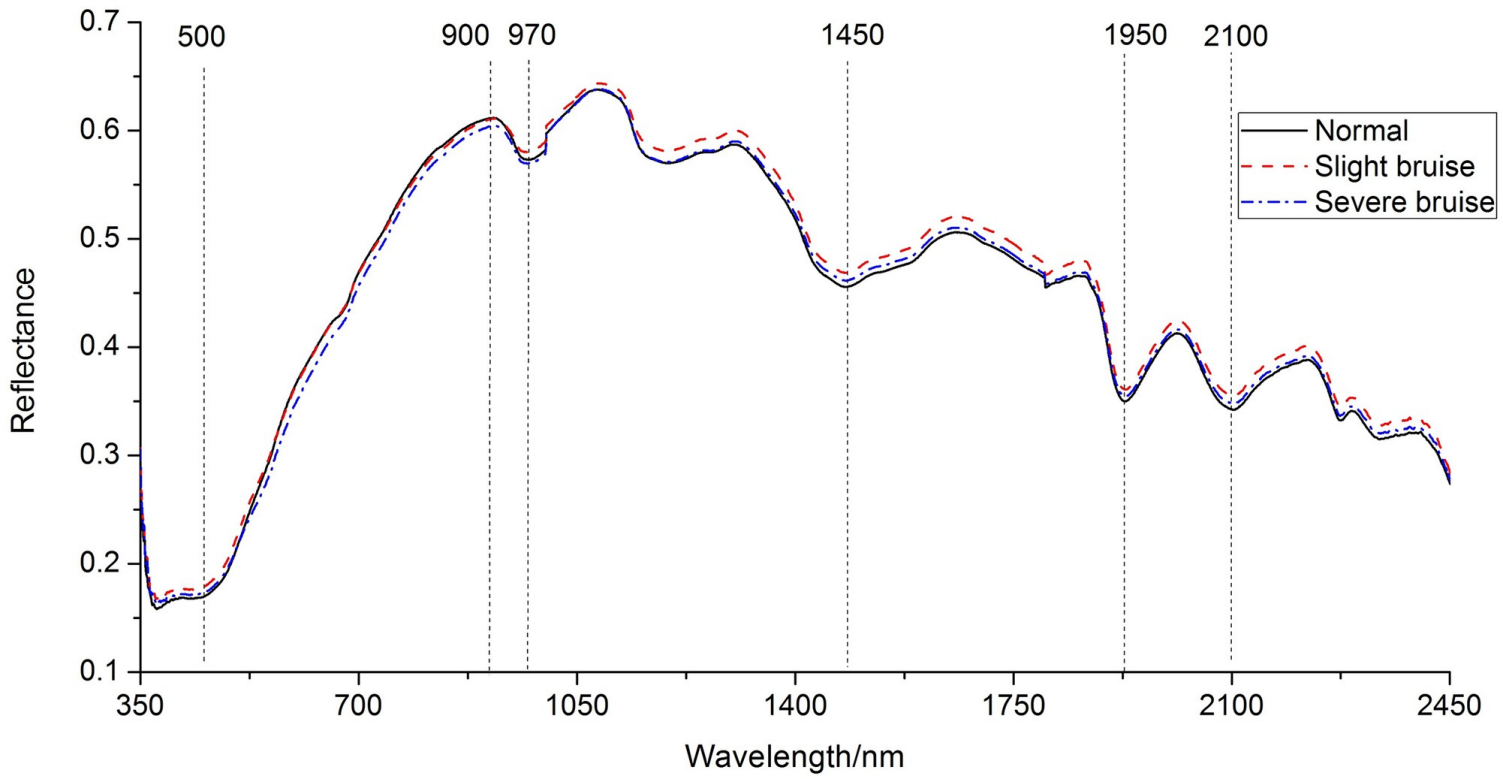
Spectral reflectance curves of the sampled ‘Huangmi’ cherry.

The mean spectral reflectance curves of ‘Huangmi’ sweet cherry covering the range of 350–2500 nm were illustrated in Fig 3. It could be observed that spectral reflectance curves for three categories of cherries showed similar profiles and trends. The spectral reflectance curves of normal and slight bruise were smooth and almost the same from 500 to 900 nm closer to visible waveband, which showed that it might be hard to classify between normal and slight bruise. The spectral reflectance of the severe bruised cherry was the lowest in the range of 500–900 nm because of colour change [54]. In other words, the visible reflectance for severe bruise cherries was the smallest among three categories of cherries because the colour of severe bruise cherries became darker and browner than those of normal and slight bruise. By contrast, spectral reflectance curves for three categories of cherries were fluctuant from 900 to 2500 nm known as NIR wavebands, the reflectance value of slight bruise was higher than the others. This was assumed to be related to firmness and solid solution content in cherry, and such phenomena was also found in pears [12, 20].

In detail, the small peak around 550 nm was associated with anthocyanin in the cherry tissue [36]. The valleys around 970 and 1450 nm, corresponding to second- and first-overtone O-H stretching [55], were related to water content in the cherries. Meanwhile, the valley around 1200 nm was assigned to the second-overtones of C-H stretching. The valleys around 1950 and 2100 nm were generally referred to O-H stretching which was caused by the combined effects of water in cherries [56].

### Principal component analysis

**Fig 4:**
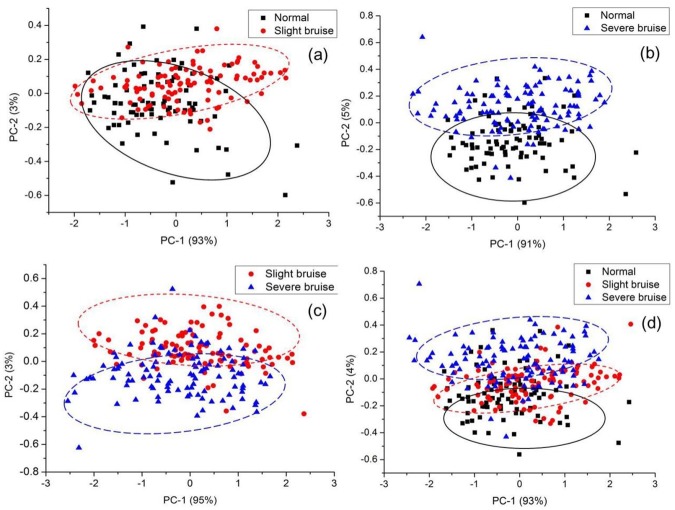
Cluster plots based on PC-1 and PC-2 for different cherry categories samples. (a) cluster plot based on PC-1 and PC-2 between normal and slight bruise; (b) cluster plot based on PC-1 and PC-2 between normal and severe bruise; (c) cluster plot based on PC-1 and PC-2 between slight and severe bruise; (d) cluster plot based on PC-1 and PC-2 between normal, slight and severe bruise.

PCA method was used for qualitative analysis of differentiating cherry bruise degrees, which was performed on the different combination of pre-processed spectra among normal, slight bruise and severe bruise cherries. The first two PCs from PCA contained most of the spectral data, and the corresponding score plots based on PC-1 and PC-2 were shown in Fig 4. In the group of normal and slight bruise, PC-1 and PC-2 explained 93% and 3% of the variations among samples, 91% and 5% in the normal and severe bruise groups, and 95% and 3% in the group of slight bruise and severe bruise, respectively. It was noticed from Fig 4(a), 4(b) and 4(c) that there were obvious differentiations in PC-2 direction from the negative side to the positive side between every two groups of cherries samples. In other words, PC-2 offered a relatively higher contribution to classify the cherry samples than PC-1.

It was also found in the Fig 4(d) that PC-1 and PC-2 explained 93% and 4% of the variations among three categories of cherries, and partly overlaps were still observed. In order to classify normal, slight and severe bruise cherries correctly, the corresponding loadings of PC-1 and PC-2 would be analyzed, and the optimal wavelengths would be selected on PC-2 by multivariate analysis methods to extract and concentrate the connotative spectra information. Furthermore, identification models were required to be investigated for qualitative analysis of cherry bruise degree.

### Optimal wavelengths selection

**Fig 5:**
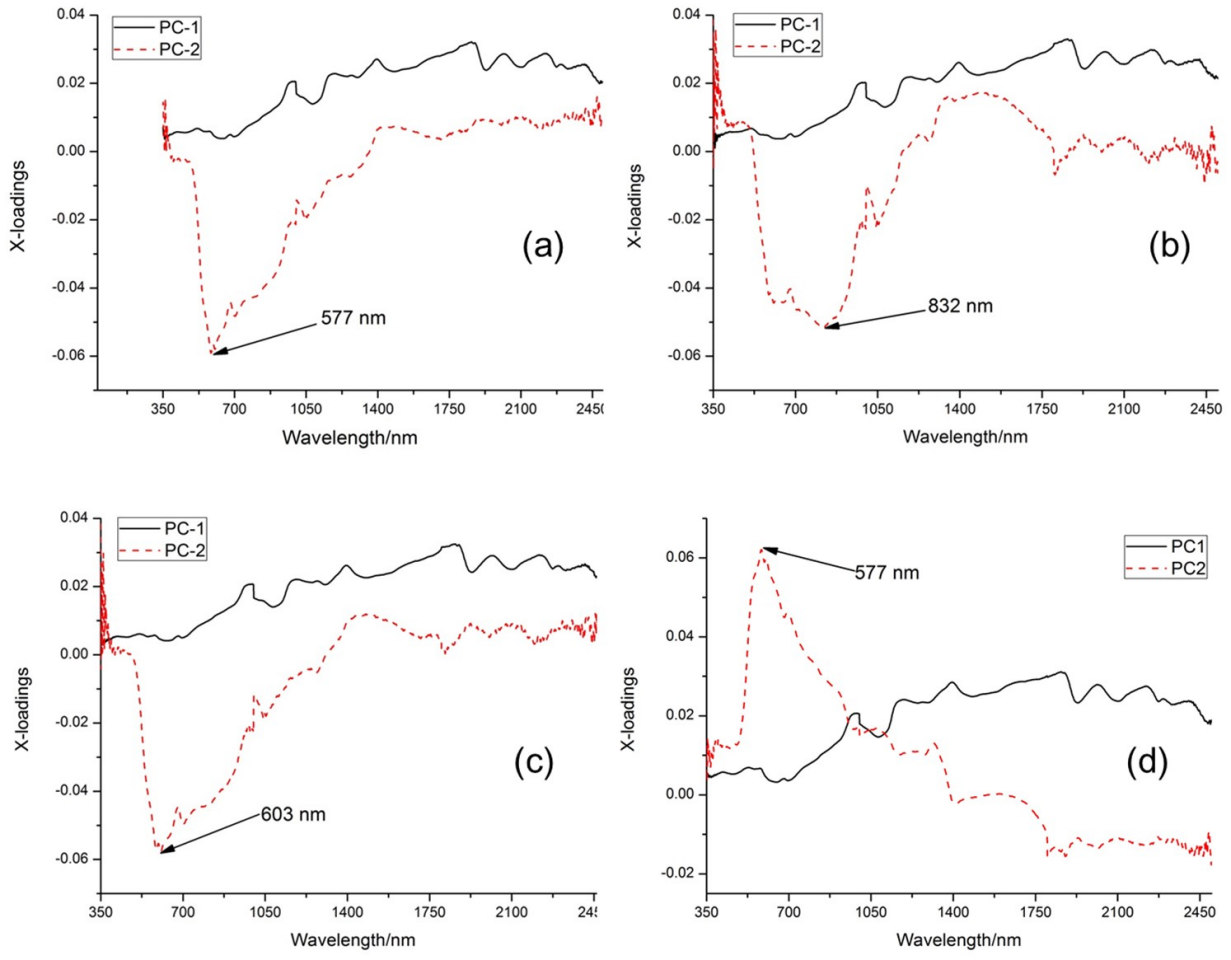
Loadings plots of the PC-1 and PC-2 for different cherry categories samples. (a) Loadings of the PC-1 and PC-2 for normal and slight bruise; (b) Loadings of the PC-1 and PC-2 for normal and severe bruise; (c) Loadings of the PC-1 and PC-2 for slight and severe bruise; (d) Loadings of the PC-1 and PC-2 for normal, slight and severe bruise.

Optimal wavelength which is particularly representative of the spectral information is selected to reduce the dimensionality and improve computational efficiency. As an effective wavelength selection method, loadings of the first two PCs were applied to identify optimal wavelengths. Fig 5 showed clearly the loading plots and the corresponding optimal wavelengths extracted from the first two PCs for three categories of cherries. In general, peaks and valleys in loading plots, which revealed the relatively high absolute loading values, were identified as the optimal wavelengths for cherry bruise discrimination. It could be observed the loading on each PC-2 had a big valleys or peaks at wavelength of 577, 832, 603, and 577 nm in Fig 5(a), 5(b), 5(c) and 5(d), respectively, and the corresponding optimal wavelengths were labelled with the arrow pointing. Therefore, the optimal wavelengths selected by analysing the loadings of PCs were 577, 603, 832 nm as shown in Table 2.

**Table 2:** LS-SVM model identification results based on optimal wavelengths and full spectra.

Wavelength selection	Optimal wavelengths	Variable	Prediction			Accuracy /%	Overall accuracy /%
			1	2	3		
	Full spectra	1	20	1	0	95.2	97.3
		2	1	24	0	96	
		3	0	0	29	100	
Loading of PCs	577, 603, 832	1	15	6	0	71.4	80
		2	7	18	0	72	
		3	0	2	27	93.1	
SPA (indirect)	365, 457, 514, 585, 606, 654	1	18	3	0	85.7	90.7
		2	3	22	0	88	
		3	0	1	28	96.6	
SPA (direct)	603, 633, 679, 1083, 1803	1	19	2	0	90.5	93.3
		2	3	22	0	88	
		3	0	0	29	100	

To compare the performance of the optimal wavelengths selection with PCA, SPA was used to select optimal wavelengths in different combination among these three categories of cherries. Five optimal wavelengths of determining the bruise degree of cherries selected from SPA were 603, 633, 679, 1083, and 1803 nm. Specifically, the optimal wavelengths selected from SPA were 457 and 606 nm between normal and slight bruise. Two optimal wavelengths including 365 and 585 nm were also identified between normal and severe bruise. For slight and severe bruise, there were two optimal wavelengths (514 and 654 nm) selected from SPA. Thus, another combination of the optimal wavelength for discriminating three categories of cherries was 365, 475, 514, 585, 606, and 654 nm by indirect SPA method. All these optimal wavelengths were shown in the second column of Table 2.

### LS-SVM classification model

Based on the optimal wavelengths selected, LS-SVM methodology was employed to establish supervised classification models for discriminating bruise degree of cherry. As shown in Table 2, classification accuracy referred to the results of prediction exercises carried out using the models to predict on the prediction sets. As a consequence of wavelengths selected by analysing the loadings of PCs, optimal wavelengths (577, 603, and 832 nm) were employed to establish the LS-SVM model for determining cherry bruise degree instead of the full spectra, and the classification accuracy being 80% as shown in Table 2. Part of the result was due to the fact the class values of cherries failed to take into account among modelling process when optimal wavelengths were selected just from loadings of PCs, producing a low classification accuracy. In contrast, LS-SVM model established on the full spectra from 350 to 2500 nm had the best prediction effect with an accuracy of 97.3%, but a similar classification accuracy of 93.3% was also achieved when LS-SVM model developed on five optimal wavelengths (603, 633, 679, 1083, and 1803 nm) selected directly by SPA, which was higher than classification accuracy of 90.7% acquired by LS-SVM combined with indirect SPA method. Further analysis of the confusion matrix shown in Table 2, it could be observed that both normal and slight bruise cherries were classified scarcely as severe bruise ones. But misclassifications would happen between normal and slight bruise cherries. The overall results showed that LS-SVM models using the optimal wavelengths selected from SPA presented better determination of bruise degree for cherry than other LS-SVM models due to its less computational load, which indicated that Vis-NIR reflection spectroscopy could be used for discriminating the bruise degree of cherry efficiently with support of multivariate analysis.

### SSC and firmness analysis

To further validate the ability of Vis-NIR spectroscopy to discriminate bruise degree of cherry, the latent relationship between spectral property and the chemical compositions of cherries in varying bruise degree was investigated, and SSC and firmness were measured after spectral data acquisition and summarized in Table 3. It could be deduced from Table 3 that the mean firmness of severe bruise was smallest and the SSC of normal samples was lowest. Considering the comprehensive influence of hardness and SSC on spectral reflectance, and consequently, the spectral curve of severe bruised cherry was closer to that of normal cherry in the NIR waveband (900–2500 nm). In three categories of cheery samples, both the firmness and SSC of slight bruise samples were highest. As a result, it has the highest reflectance from 900 nm to 2500 nm shown in Fig 3. Also, further research could be focused on exploring the relationship between the chemical value and those wavelengths [21, 44].

**Table 3:** Statistic data of firmness and SSC in cheery samples.

Samples indexes	Firmness (kg/cm2)				SSC (°Brix)			
	Maximum	Minimum	Mean	SD*(%)	Maximum	Minimum	Mean	SD*(%)
Normal	4.66	2.85	3.81	0.462	16.9	13	15.26	1.207
Slight bruise	5.6	3.11	4.12	0.583	18.4	14	15.81	1.138
Severe bruise	4.43	2.07	3.07	0.63	19.5	12.6	15.33	1.991

Note:

*SD = Standard deviation.

## Conclusion

Bruise identification was very helpful in sorting cherry in accordance with its bruise degree. This study demonstrated the high potential of Vis-NIR reflection spectroscopy coupled with multivariate analysis for determination of cherry bruise degree. LS-SVM model developed using the optimal wavelengths selected directly by SPA showed better performance for identifying bruise degree of cherry with an accuracy of 93.3%, which was practical due to its less computational load. Whereas the LS-SVM model developed using full spectra achieved a similar predictive accuracy of 97.3%, and misclassification generally occurred in normal and slight bruise cherry samples. Furthermore, the latent relationship between spectral property in varying bruise degree of cherries and SSC and firmness was investigated, and both exterior attributes and internal quality were consistent with the Vis-NIR reflection spectroscopy of cherry. This study was limited to spectral information, therefore in the future work, hyperspectral imaging will be employed in fruit bruise degree determination, which might provide more satisfactory recognition performance by the image integrating both the spectral information and spatial information. In particular, on-line sensors will also be developed for cherry sorting or post-harvest quality processing of other fruits.
